# Isolation and Characterization of the Adamantinomatous Craniopharyngioma Primary Cells with Cancer-Associated Fibroblast Features

**DOI:** 10.3390/biomedicines13040912

**Published:** 2025-04-09

**Authors:** Dongting Chen, Ting Lei, Yulin Wang, Zaitao Yu, Siqi Liu, Ling Ye, Wanfang Li, Qin Yang, Hongtao Jin, Fangjun Liu, Yan Li

**Affiliations:** 1 Beijing Key Laboratory of New Drug Mechanisms and Pharmacological Evaluation Study, Department of Pharmacology, Institute of Materia Medica, Chinese Academy of Medical Sciences and Peking Union Medical College, Beijing 100050, China; chendt@imm.ac.cn (D.C.);; 2 Department of Neurosurgery, Sanbo Brain Hospital, Capital Medical University, Beijing 100093, China; leiting@mail.ccmu.edu.cn (T.L.);; 3 New Drug Safety Evaluation Center, Institute of Materia Medica, Chinese Academy of Medical Sciences & Peking Union Medical College, Beijing 100050, China; 4 Beijing Union-Genius Pharmaceutical Technology Development Co., Ltd., Beijing 100176, China

**Keywords:** neuro-oncology, craniopharyngioma, cancer-associated fibroblast features, primary cell, transcriptional signature

## Abstract

**Backgrounds**: Adamantinomatous craniopharyngiomas (ACPs) are benign intracranial tumors that behave aggressively due to their location, infiltration of the surrounding nervous tissue and high capacity for recurrence. In this study, we aimed to construct ACP primary cell models for further investigation of tumorigenic and recurrent mechanisms. **Methods**: Primary cells were isolated from primary (one case) and recurrent (one case) ACP. Short tandem repeat (STR) analysis was used to clarify the identity of the ACP primary cells we isolated. Whole exome sequencing (WES), immunofluorescence (IF) and immunohistochemistry (IHC) were performed on primary cells and corresponding ACP tissues, to determine the mutational profile and to clarify the tissue origin and phenotypic of primary cells. Transcriptome RNA-seq was performed to obtain the gene expression characteristics of ACP primary cells. Subsequently, a heterotopic ACP xenograft mouse model was established to confirm the tumorigenesis capacity of ACP primary cells. **Results**: ACP primary cells were successfully cultured. The genetic variants were similar to the original tumor tissue, and they owned expression of cancer-associated fibroblast (CAF) markers (FSP1/S100A4, Vimentin) and nuclear translocation β-catenin. Meanwhile, they had an high level expression of extracellular matrix components (Fibronectin). The tumor formation ability of ACP primary cells was verified. The transcriptional signatures of ACP primary cells were also explored. **Conclusions**: We successfully isolated and characterized ACP primary cells that acquired multiple CAF features and demonstrated stable propagation through dozens of passages. These PDC models laid the foundation for further research on ACP.

## 1. Introduction

Adamantinomatous craniopharyngiomas (ACPs) are rare intracranial tumors that occur either in the sella turcica (intrasellar) or above the sella turcica (suprasellar) [1]. It is generally believed that ACPs originates from the remnants of the craniopharyngeal duct epithelium [1,2]. Epidemiological data show that ACPs have a bimodal peak distribution of onset age in children aged 5–15 years and adults aged 45–60 years [3,4]. Although ACPs have a low histological grade [5], they are still a challenge to treat due to their close proximity to neurovascular structures [6]. Moreover, tumor epithelia with finger-like protrusions exhibit invasive properties [1,7,8], causing endocrine deficiencies and hypothalamic syndrome. Meanwhile, oncothlipsis causes progressive visual impairment [9,10]. Surgical treatment is still the first-line treatment for ACPs [1], but it is difficult to completely remove the lesions during surgery [5]. Gross total resection (GTR) effectively decreases the recurrence rate, but it still remains relatively high, ranging from 11.1% to 25% [11].

It is generally believed that ACPs are driven by somatic mutations in CTNNB1 within exon 3 [12,13], which leads to the activation of the Wnt/β-catenin signaling pathway [14]. Previous studies have described the diversity of ACP tissue structures and cell types [2,15]. The solid part of the tumor includes palisade-like epithelial cell layers, helical cell clusters, keratin nodules and stellate reticulum [1]. Reactive gliosis and connective tissue were also present adjacent to the tumor. More attention should be given to the intracellular regulatory networks due to their crucial role in the tumorigenesis, progression and invasion of ACP [16]. A deeper look at the development and recurrence mechanism of ACP is needed to identify potential therapeutic targets.

Cell and cell-derived subcutaneous tumor models are basic and efficient tools for preclinical cancer research [17,18]. Several studies have reported the establishment of PDC, PDO, PDX and spontaneous animal models of ACPs. The studies by Wang and colleagues have cultured the epithelioid ACP primary cells and established a TERT-transfected immortalized ACP cell line STAM4 [19,20]. Tang et al. have established the 3D organoid model of ACPs that is used for the evaluation of targeted immunotherapy [21]. Annett et al. have characterized the murine orthotopic ACP PDX model [22]. Moreover, Juan Pedro’s team have reported the Sox2CreERT2/+; Ctnnb1lox(ex3)/+ mice and Hesx1Cre/+; Ctnnb1+/lox(ex3) mice developed pituitary tumors that resemble human ACP [23,24].

In this study, we aimed to construct ACP primary cell models for further investigation of tumorigenic and recurrent mechanisms. The ACP primary cells with CAF features were successfully cultured from primary and recurrent ACPs, and a series of experiments were conducted to verify the tissue origin, phenotypic characteristics and tumorigenicity of the primary cells. The use of ACP primary cells may provide additional opportunities for new insights and discoveries in research about ACPs.

## 2. Materials and Methods

### 2.1. ACP Clinical Samples

Patients with primary or recurrent ACP who underwent surgery at Sanbo Brain Hospital, Capital Medical University, Beijing, China, and received pathological confirmation of ACPs were enrolled in this study. None of the participants received radiotherapy, chemotherapy, or any other form of antitumor therapy before surgery. Informed consent was obtained from all participants or their parents or legal guardians. The study was approved by the Institutional Review Board of Sanbo Brain Hospital (Approval NO. SBNK-YJYS-2023-031-01). All methods were performed in accordance with the guidelines of the Institutional Review Board of Sanbo Brain Hospital and the Declaration of Helsinki.

### 2.2. ACP Primary Cell Culture

One case of primary ACP (0913) and one case of recurrent ACP (0824) were used for cell isolation. The tumor specimens were stored in sterile tubes at 4 °C and sent for primary cell culture immediately after excision surgery at Sanbo Brain Hospital. After clearing the blood from the tumor tissues, the tissues were dissociated into single-cell suspensions using a Human Tumor Dissociation Kit (Miltenyi Biotec, Bergisch Gladbach, Germany). Subsequently, phosphate-buffered saline (PBS) was added to the cell suspension to terminate the digestion, the suspension was centrifuged at 1000 rpm for 5 min to collect the cells, and the cells were washed 2 times with PBS buffer to fully remove the digestion medium. Finally, the ACP primary cells were resuspended with culture medium: DMEM Nutrient Mix F12 (Thermo Fisher Scientific, Waltham, MA, USA) supplemented with 5% fetal bovine serum (YuanHeng ShengMa Biology Technology Research Institute, Beijing, China), 20 ng/mL Human EGF Protein (Sino Biological, Beijing, China). The ACP primary cell cultures were maintained in a humidified 5% CO_2_ incubator (Thermo Fisher Scientific, Waltham, MA, USA) at 37 °C and passaged at a density close to 80%.

### 2.3. Mouse Xenograft Model

Specified pathogen-free male NCG mice (aged 8 weeks) were purchased from Jiangsu GemPharmatech LLC. (Nanjing, China). Cultured ACP primary cells were digested, collected in PBS and seeded in the right flank area by subcutaneous injection. Each mouse was seeded with 5 × 10^5^ primary cells embedded in Matrigel (Corning, Corning, NY, USA), 0824 and 0913 primary cells were passaged for 7 passages at the time of inoculation, each cell strain was inoculated in one mouse. 0824 and 0913 primary cells continued to grow in mice for 14 and 11 weeks, respectively. Then, the mice were sacrificed by cervical dislocation based on touchable palpable mass subcutaneous, and the xenograft tumors were removed for subsequent experiments.

### 2.4. H&E Staining

The tumor tissues from patients or model mice were fixed in 10% neutral buffered formalin and embedded in paraffin. The tumor tissue sections were heated at 64 °C for 1 h in an oven, deparaffinized with xylene, and rehydrated with an ethanol gradient (100–50%). Next, the sections were stained with hematoxylin solution for 5 min, dipped in 1% acid ethanol (1% HCl in 70% ethanol), and then rinsed with distilled water. Then, the sections were stained with eosin solution for 3 min, dehydrated with ethanol, and cleared in xylene. Representative images were obtained by light microscopy.

### 2.5. Immunofluorescence Staining

Paraffin-embedded tumor tissue sections from patients or model mice were subjected to deparaffinization and rehydration, and antigen retrieval was performed by heating the sections in Tris-EDTA solution (pH 9.0) or sodium citrate buffer (pH 6.0) in a pressure cooker for 3 min. Subsequently, the sections were incubated with blocking solution containing 10% normal goat serum (Solarbio, Beijing, China) in PBS for 1 h, followed by overnight incubation with primary antibodies in a humid chamber at 4 °C. The incubation for the negative control sections were replaced by the blocking solution. The next day, the sections were incubated with the corresponding fluorochrome-conjugated secondary antibodies in the dark at room temperature for 1 h. After the sections were covered with an anti-fluorescence attenuation sealant containing DAPI (Solarbio, China), representative images were captured by Olympus IX70 fluorescence microscopy (Olympus, Tokyo, Japan). The ACP primary cells were grown on coverslips and fixed with methanol at −20 °C for 10 min, and the tissue sections were stained. The primary and secondary antibodies used were as follows: pan-CK mouse mAb (Abcam, Shanghai, China, ab7753, 1:500); EpCAM mouse mAb (CST, Danvers, MA, USA, #2929, 1:800); FSP1/S100A4 rabbit mAb (ABclonal, Woburn, MA, USA, A19109, 1:100); Vimentin rabbit mAb (CST, #5741T, 1:200); β-catenin rabbit mAb (ABclonal, A19657, 1:100); α-SMA rabbit mAb (CST, #19245, 1:200); Fibronectin rabbit mAb (ABclonal, A23830, 1:1000); ABflo 488-conjugated goat anti-rabbit IgG (ABclonal, AS053, 1:200); and ABflo 594-conjugated goat anti-mouse IgG (ABclonal, AS054, 1:200).

### 2.6. Immunohistochemical Staining

Paraffin-embedded tumor tissue sections from patients were deparaffinated and rehydrated. Endogenous peroxidase was inactivated with 3% H_2_O_2_ for 20 min at room temperature. After antigen retrieval in Tris-EDTA solution (pH 9.0) or sodium citrate buffer (pH 6.0) and blocking, the sections were incubated with pan-CK mouse mAb (Abcam, ab86734, 1:250); EpCAM mouse mAb (CST, #2929, 1:500); β-catenin rabbit mAb (ABclonal, A19657, 1:100); FSP1/S100A4 rabbit mAb (ABclonal, A19109, 1:100); Fibronectin rabbit mAb (ABclonal, A23830, 1:1000) overnight at 4 °C (The incubation for the negative control sections were replaced by the blocking solution) and HRP-conjugated anti-rabbit secondary antibody (Beyotime, Wuxi, China, P0615) at room temperature for 1 h. Protein expression was visualized using diaminobenzidine staining (Beyotime, P0202).

### 2.7. Whole Exome Sequencing

The whole exome sequencing (WES) analysis of tumor tissue FFPE samples from patients was performed by Beijing Novogene Science and Technology Co., Ltd. (Beijing, China). Genomic DNA samples were extracted. The exome sequences were efficiently enriched from 0.4 μg genomic DNA using Agilent SureSelect Human All Exon V6 (Agilent, Palo Alto, CA, USA, Catalog: 5190-8864). The detailed method of WES analysis was provided in the Supplementary Information (Document S1).

### 2.8. Short Tandem Repeat Analysis

The short tandem repeat (STR) analysis of ACP primary cells was performed by Wuhan Procell Life Technology Co., Ltd. (Wuhan, China). In brief, an appropriate amount of 0913 or 0824 ACP primary cells (about 1 × 10^6^ cells, G6) were used TIANamp Genomic DNA Kit (TIANGEN, Beijing, China) to extract DNA, 20 STR loci and gender identification loci were amplified by MicroreaderTM21 ID System, PCR product detection was performed by GenReader 7010 genetic analyzer (Microread, Beijing, China), detection results were analyzed by GeneMapper Software6 (Thermo Fisher Scientific, Waltham, MA, USA), and compared with ExPASy database (https://web.expasy.org/cellosaurus-str-search/, accessed on 13 January 2024). Data sources included ATCC, DSMZ, JCRB and other cell banks, as well as literatures.

### 2.9. Transcriptome Sequencing

Transcriptome RNA-seq of 0913 or 0824 ACP primary cells (about 1 × 10^6^ cells, G6) was performed by Beijing Novogene Science and Technology Co., Ltd. RNA integrity was assessed using the RNA Nano 6000 Assay Kit of the Bioanalyzer 2100 system (Agilent Technologies, USA). Total RNA was used as input material for the RNA sample preparations, PCR products were purified on an AMPure XP system (Beckman Coulter, Brea, CA, USA), and library quality was assessed on an Agilent Bioanalyzer 2100 system. Clustering of the index-coded samples was performed on a cBot Cluster Generation System using the TruSeq PE Cluster Kit v3-cBot-HS (Illumina, San Diego, CA, USA) according to the manufacturer’s instructions. After cluster generation, the library preparations were sequenced on an Illumina NovaSeq platform, and 150 bp paired-end reads were generated.

### 2.10. Bioinformatics Analysis

The index of the reference genome was built using HISAT2 v2.0.5, and the clean reads were aligned to the reference genome using HISAT2 v2.0.5. FeatureCounts v1.5.0-p3 was used to count the number of reads mapped to each gene. RNA-seq data for three groups of normal brain tissues (glioma adjacent tissues) were obtained from The Cancer Genome Atlas (TCGA) (https://www.cancer.gov/ccg/research/genome-sequencing/tcga, accessed on 18 April 2024). Differential expression analysis between ACP primary cells and normal tissues was performed using the DESeq2 R package (1.20.0). Gene Ontology (GO) enrichment analysis, Kyoto Encyclopedia of Genes and Genomes (KEGG) pathway enrichment analysis, and gene set enrichment analysis (GSEA) of the differential expressed genes (DEGs) were performed via an online bioinformatics analysis platform (https://www.bioinformatics.com.cn/, accessed on 18 April 2024; https://cmb.bnu.edu.cn/imm/index.php/trans, accessed on 18 April 2024) and GSEA (v4.3.3) software. GATK (v4.1.1.0) software was used to perform single-nucleotide polymorphism (SNP) calling, and SnpEff (4.3.1q) software was used for mutation site annotation.

## 3. Results

### 3.1. Isolation and Culture of ACP Primary Cells

**Figure 1:**
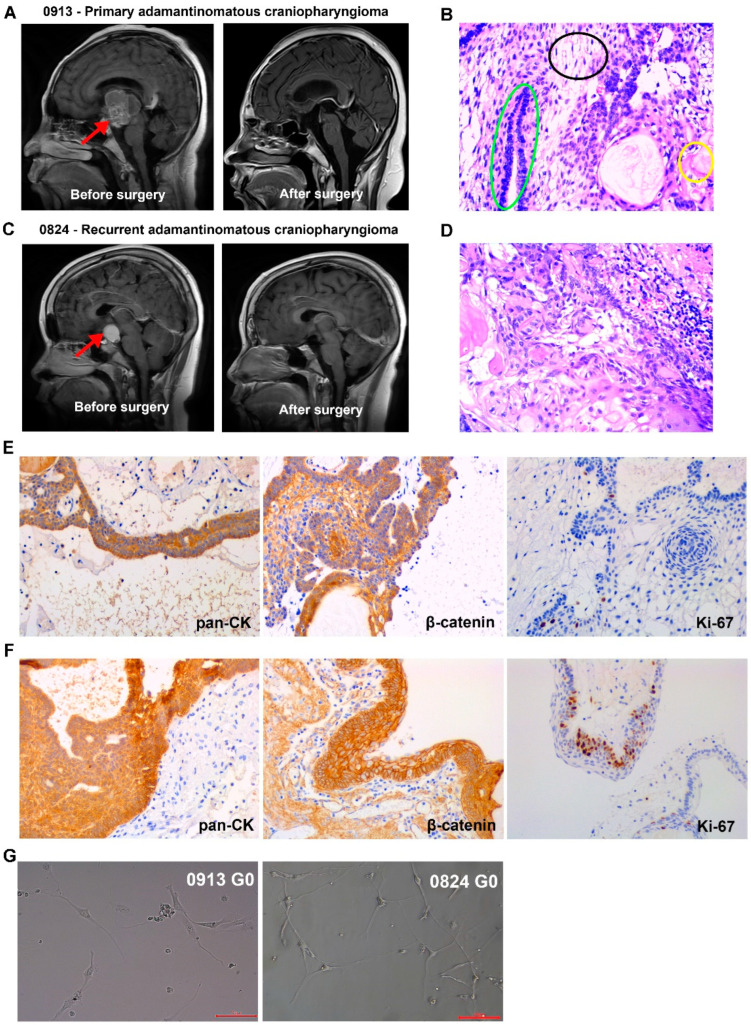
Clinical, pathological and primary cell images of adamantinomatous craniopharyngiomas (ACPs). (**A**) Representative brain magnetic resonance images of primary ACP patient 0913 before and after surgery. Red arrows indicate tumor lesions. (**B**) Representative H&E-stained image of tumor tissue sections from patient 0913. Green circle: palisade-like epithelial cell layer; black circle: stellate reticulum; yellow circle: keratin nodules. (**C**) Representative brain magnetic resonance images of recurrent ACP patient 0824 before and after surgery. Red arrows indicate tumor lesions. (**D**) Representative H&E-stained image of tumor tissue sections from patient 0824. (**E**) Immunohistochemical staining of 0913 tumor tissue. (**F**) Immunohistochemical staining of 0824 tumor tissue. (**G**) Representative reflection microscopy images of cultured 0913 and 0824 ACP primary cells at 0 generation; 200×; red scale bar: 100 μm.

Patients 0913 and 0824, who were diagnosed with primary and recurrent ACP, respectively, were enrolled in this study. Representative magnetic resonance images for these patients are shown in Figure 1A,C. The typical organizational structures of the ACP could be observed in tumor tissue (Figure 1B,D). The IHC results revealed that pan cytokeratin (pan-CK) and β-catenin were all positive in tumor tissues from patients 0913 and 0824, and β-catenin nuclear translocation was observed (Figure 1E,F). BRAFV600E was negative (Table 1).

ACP primary cells were isolated from parental tumor tissues and cultured, and all of the cells were passaged for more than 10 generations. Representative reflection microscopy images of 0913 and 0824 primary cells at 0 generation were shown in Figure 1G. Both 0913 and 0824 primary cell lines showed a spindle pattern at the 0 generation of culture, which were similar to the CAF characteristic.

**Table 1:** Clinical and pathological characteristics of the patients with adamantinomatous craniopharyngiomas (ACPs).

Patients	Age (Years)	Sex	Pathological Diagnosis	TUMOR Morphology	IHC			
					pan-CK	β-Catenin	BRAFV600E	Ki-67
0913	19	female	primary ACP	cystic degeneration, calcification	+	+	−	+2%
0824	10	female	recurrent ACP	cystic degeneration, calcification, ossification, cholesterol crystal	+	+	−	+15%

### 3.2. Short Tandem Repeat Analysis

The STR typing results indicated that no cross contamination of human cell lines was found in the 0913 and 0824 ACP primary cells. The genotyping results of the STR loci of the 0913 and 0824 ACP primary cells are shown in Table 2. A low degree of matching could be detected between cultured ACP primary cells and the cell line data in the cell bank, and the highest matching degree was only 78.57%, suggesting that the gene locus information for the 0913 and 0824 ACP primary cells has not been included in the existing cell banks.

### 3.3. Identification of ACP Primary Cells with CAF Features

To clarify the tissue origin of the ACP primary cells, we performed WES analysis of the gene mutations in the original tumor tissue and RNA-seq to analyze the single nucleotide polymorphisms (SNPs) of the corresponding primary cells. The average effective data proportion of WES was 96.24%, the average Q20 and Q30 values were 98.05% and 94.70%, respectively, and the average error rate was 0.03%. In general, the quality of the sequencing data was good and met the analytical requirements. We compared the mutations of the significantly mutated genes (SMGs) and known driver genes in the parental tissues with those in the corresponding primary cells. The results showed that 0913 tissue had a high frequency of missense mutations in CTNNB1 in exons 3 and 4 of chromosome 3, but this mutation was not found in 0913 primary cells, whereas mutations of varying degrees and effects on ALDH16A1, ACSL3 and COL3A1 were found in both 0913 tissue and 0913 primary cells (Table 3a). In 0824 tissue, the same missense mutation in CTNNB1 was detected; moreover, we detected a synonymous mutation in exon 15 of chromosome 3. This CTNNB1 synonymous mutation was also found in 0824 primary cells, and coincident mutations in UBE4A, ALDH16A1 and SLC12A8 were found in both 0824 tissue and 0824 primary cells (Table 3b). Based on these results, we concluded that the 0913 and 0824 primary cells possessed homology with the corresponding clinical samples.

**Figure 2:**
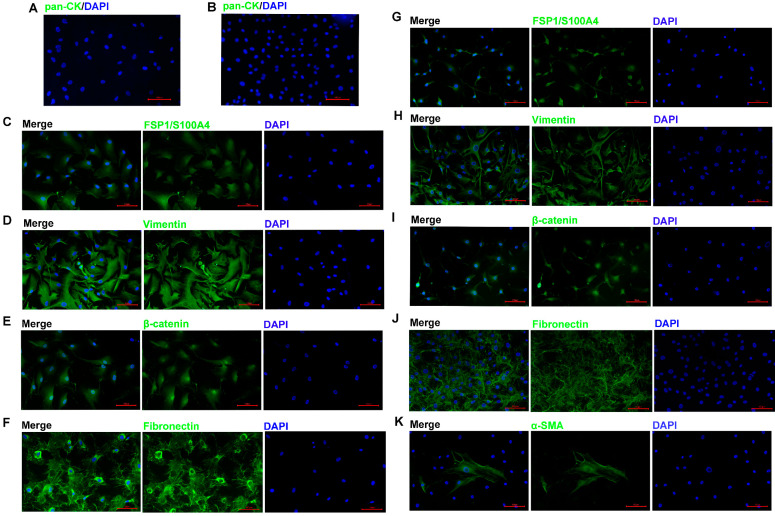
ACP primary cells identification by immunofluorescence. Immunofluorescence staining of 0913 primary cells, representative images of pan-CK (**A**), FSP1/S100A4 (**C**), Vimentin (**D**), β-catenin (**E**) and Fibronectin (**F**) staining are shown. Immunofluorescence staining of 0824 primary cells, representative images of pan-CK (**B**), FSP1/S100A4 (**G**), Vimentin (**H**), β-catenin (**I**), Fibronectin (**J**) and α-SMA (**K**) staining are shown; 200×; red scale bar: 100 μm.

To reveal the phenotypic characteristics of the ACP primary cells we isolated, IF staining was performed to identify the cell type. The results showed that pan-CK expression was negative in 0913 and 0824 primary cells (Figure 2A,B), and EpCAM expression was also negative. The expression of CAF markers FSP1 (Figure 2C,G) and Vimentin (Figure 2D,H) was all positive in 0913 and 0824 primary cells; however, positive staining for α-SMA was observed only in a few individual cells with larger cell bodies (Figure 2K). Meanwhile, strong positive staining for Fibronectin was observed in the two primary cell lines (Figure 2F,J). These results demonstrated that the primary cells isolated from primary and recurrent ACP tumor tissues all obtained CAF characteristics. Notably, nucleus translocation of the β-catenin, the marker of the ACP, was observed in both 0913 and 0824 primary cells (Figure 2E,I). Therefore, ACP primary cells with CAF features were successfully isolated and cultured.

### 3.4. ACP Primary Cells with CAF Features Derived from ACP Tumor Tissues

**Figure 3:**
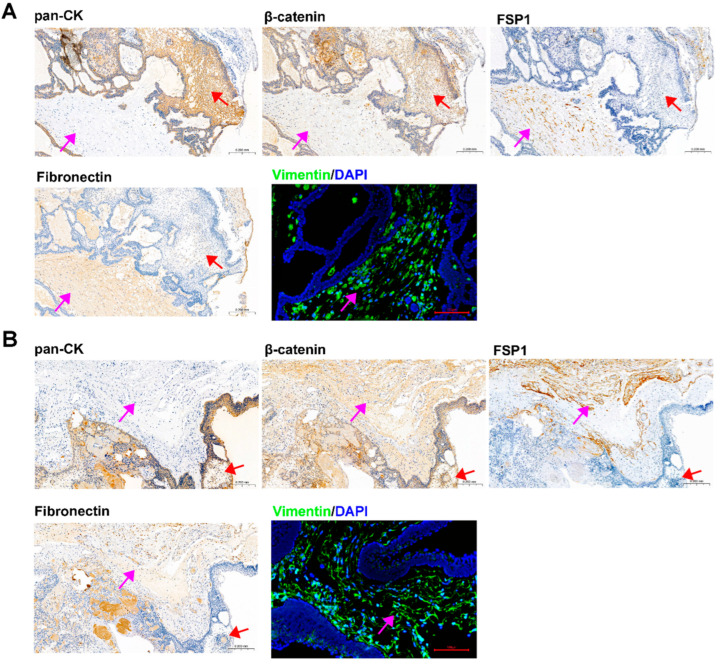
Identification of ACP tumor tissue sections from patients 0913 and 0824. (**A**) Immunohistochemical and immunofluorescence staining of tumor tissue sections from patient 0913, representative images of pan-CK, β-catenin, FSP1/S100A4, Fibronectin and Vimentin are shown. (**B**) Immunohistochemical and immunofluorescence staining of tumor tissue sections from patient 0824 and representative images of pan-CK, β-catenin, FSP1/S100A4, Fibronectin and Vimentin are shown. IHC figure at 150×, black scale bar: 200 μm; IF figure at 200×, red scale bar: 100 μm. Pink arrow: fibrous stroma area; red arrow: palisade-like epithelial cell layer, helical cell clusters, keratin nodules and stellate reticulum.

IHC and IF staining of serial slices of ACP tissue were performed to confirm the origin and tissue localization of primary cells. The staining results of 0913 and 0824 tissues were shown in Figure 3A,B, respectively. In 0913 tissue sections, pan-CK expression was displayed within the palisade-like epithelial cell layer, helical cell clusters, keratin nodules and stellate reticulum (marked with a red arrow), and were absent in the fibrous stroma area (marked with a pink arrow). Positive staining of β-catenin was observed in whole section, but the nuclear translocation was only presented in helical cell clusters. In contrast, strong and dispersive FSP1, Fibronectin and Vimentin expressions were only detected in the fibrous stroma area (marked with a pink arrow). A similar expression pattern was observed in the 0824 tissue sections. These staining results of primary cells (Figure 2) combined with parental tissues indicated that the isolated ACP primary cells with CAF features might be derived from the fibrous stroma of ACP.

### 3.5. Mouse Subcutaneous Xenograft Formation of ACP Primary Cells

**Figure 4:**
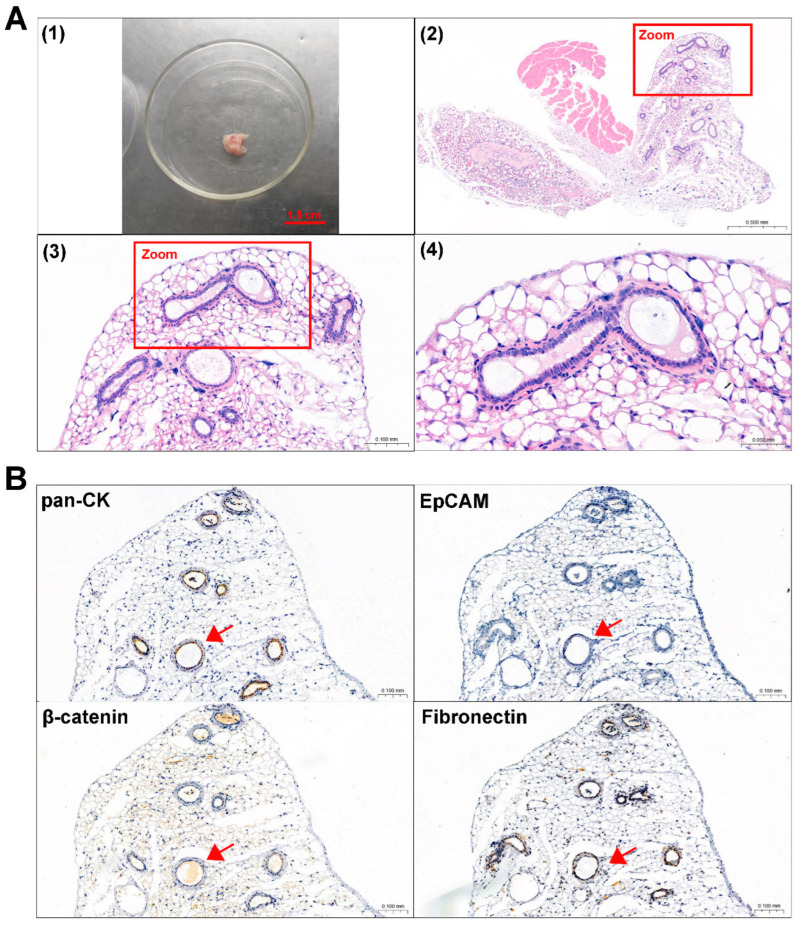
Identification of mouse subcutaneous xenograft tumors derived from ACP 0913 primary cells. (**A**) Image of the 0913 primary cell mouse xenograft tumor (**1**) and representative H&E-stained images of 0913 xenograft tumor tissue sections (**2**–**4**). The boxed areas are enlarged and indicate palisade-like epithelial cell layers. Image (**2**) at 80×, black scale bar: 0.5 mm; image (**3**) at 300×, black scale bar: 0.1 mm; image (**4**) at 600×, black scale bar: 0.05 mm (**B**) Immunohistochemical staining of 0913 xenograft tumor tissue sections, and representative images of pan-CK, EpCAM, β-catenin and Fibronectin are shown. Red arrows indicate typical palisade-like epithelial cell layers. Images at 200×, black scale bar: 0.1 mm.

**Figure 5:**
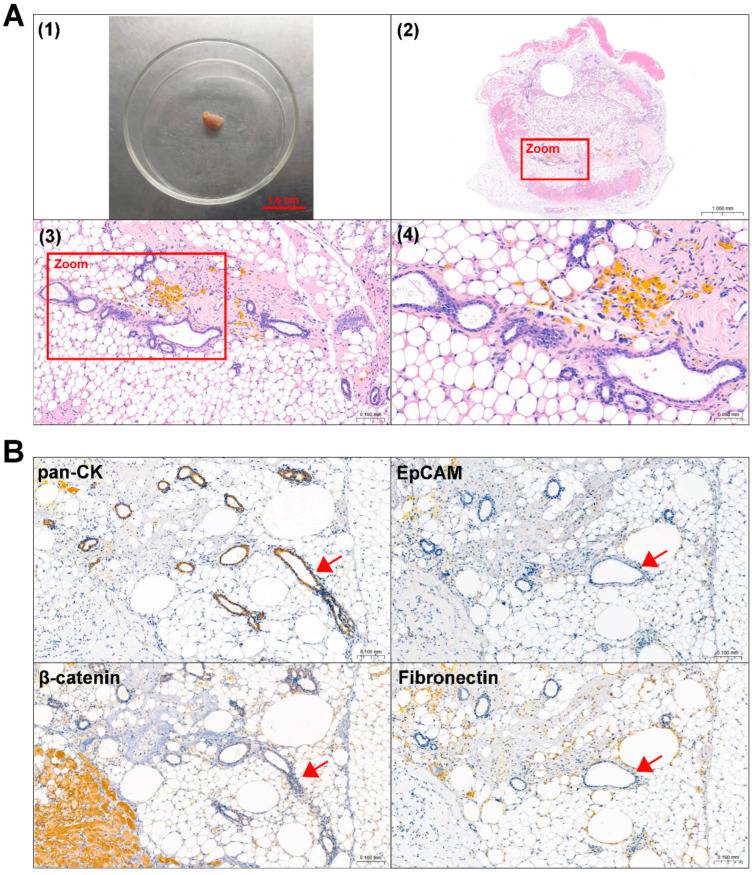
Identification of mouse subcutaneous xenograft tumors derived from ACP 0824 primary cells. (**A**) Image of the 0824 primary cell mouse xenograft tumor (**1**) and representative H&E-stained images of 0824 xenograft tumor tissue sections (**2**–**4**). The boxed areas are enlarged; they indicate palisade-like epithelial cell layers. Image (**2**) at 30×, black scale bar: 1.0 mm; image (**3**) at 200×, black scale bar: 0.1 mm; image (**4**) at 400×, black scale bar: 0.05 mm (**B**) Immunohistochemical staining of 0824 xenograft tumor tissue sections, representative images of pan-CK, EpCAM, β-catenin and Fibronectin are shown. Red arrows indicate typical palisade-like epithelial cell layers. Images at 200×, black scale bar: 0.1 mm.

Primary 0913 and 0824 cells were transplanted subcutaneously into NCG mice to investigate tumorigenicity. A period of time after cell injection, the formation of tumors was observed in the 0913 and 0824 primary cell xenograft models (Figure 4A and Figure 5A). H&E staining was performed to confirm the histological properties of the xenografts. These scattered epithelium structures (red boxed areas) which had hyperchromatic nuclei were similar to the palisading epithelium structures in ACP. Immunohistochemical staining of serial slices of 0913 and 0824 xenograft tissue was performed to further identify the phenotypic characteristics of these structures (Figure 4B and Figure 5B). As the images showed, pan-CK (anti-human) was strongly positive in the palisade-like epithelial cell layers, accompanied by weaker positive staining of β-catenin, while another epithelial marker, EpCAM, was negative. The protein expression signatures described above were highly coincident with the palisading epithelium structures in human ACP tumor tissues. Meanwhile, Fibronectin expression was also observed in epithelial cell layers within 0913 xenograft tissue. Therefore, these results demonstrated that isolated ACP primary cells with CAF features could form tumors that closely mimic the characteristic histopathological features observed in patient-derived ACP specimens.

### 3.6. Transcriptional Characteristics of ACP Primary Cells

**Figure 6:**
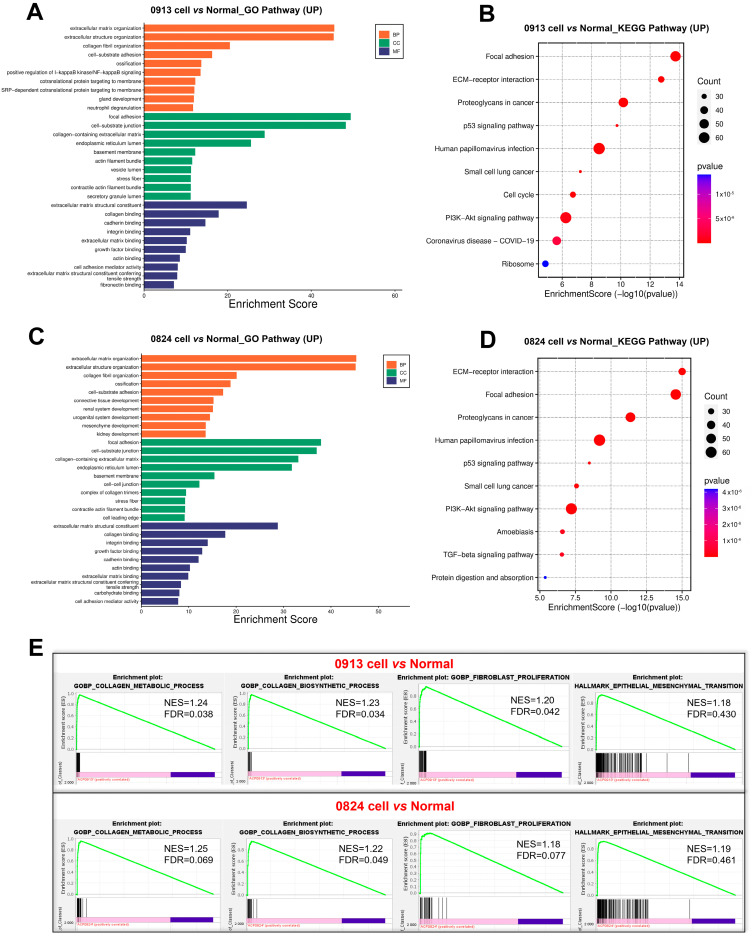
Transcriptional differences between the 0913 and 0824 primary cells with normal brain tissue. GO (**A**) and KEGG (**B**) enrichment of upregulated pathways in 0913 primary cells vs. normal tissue. GO (**C**) and KEGG (**D**) enrichment of upregulated pathways in 0824 primary cells vs. normal tissue. (**E**) Gene set enrichment analysis (GSEA) of the collagen metabolic process, collagen biosynthetic, fibroblast proliferation and epithelial mesenchymal transition pathways between the 0913 and 0824 primary cells with normal tissue.

RNA-seq was performed to better understand the gene transcriptional signatures of ACP primary cells with CAF features. Enrichment analysis of the DEGs was performed to identify the pathways associated with the DEGs between the 0913 or 0824 primary cells and normal brain tissues. GO enrichment revealed consistent upregulation of biological processes, including extracellular matrix or structure organization, collagen fibril organization, ossification and cell−substrate adhesion, in 0913 or 0824 primary cells compared with normal brain tissues (Figure 6A,C). These pathways were closely related to the properties and functions of CAF. Similar pathways were upregulated in 0913 and 0824 primary cells by KEGG analysis, including focal adhesion and ECM-receptor interaction. Moreover, proteoglycans in cancer, the p53 signaling pathway, PI3K-Akt signaling pathway and other pathways were also upregulated, as determined by KEGG analysis (Figure 6B,D). GSEA also demonstrated the upregulation of the collagen metabolic process, collagen biosynthetic, fibroblast proliferation and epithelial mesenchymal transition pathways in 0913 and 0824 primary cells (Figure 6E).

**Figure 7:**
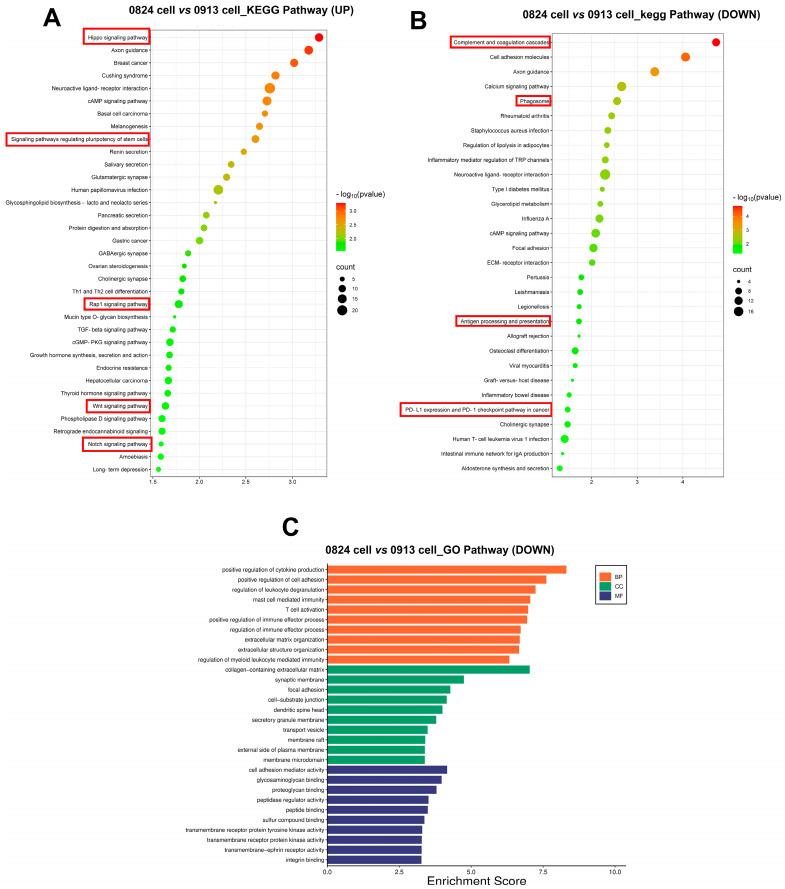
Transcriptional differences between the 0824 primary cells with 0913 primary cells. (**A**) KEGG enrichment of upregulated pathways in 0824 primary cells vs. 0913 primary cells. KEGG (**B**) and GO (**C**) enrichment of downregulated pathways in 0824 primary cells vs. 0913 primary cells.

To further explore the differences of ACP primary cells derived from primary and recurrent tumor, DEGs were calculated between 0913 and 0824 primary cells. Notably, the Hippo, Wnt, Rap1, and the Notch signaling pathways were all upregulated in 0824 primary cells (recurrent), and the signaling pathways regulating pluripotency of stem cells were also upregulated (Figure 7A). In contrast, the pathways correlated with immune system activation, such as regulation of leukocyte degranulation, mast cell-mediated immunity, and T-cell activation, were downregulated in the 0824 primary cells according to the GO analysis (Figure 7C). Moreover, the downregulation of immune-related pathways, including complement and coagulation cascades, phagosome, antigen processing and presentation, PD-L1 expression and PD-1 checkpoint pathway in cancer, was also found in the KEGG enrichment analysis of the 0824 cells (Figure 7B).

## 4. Discussion

Despite continuous progress in neurosurgery techniques, ACPs remain challenging tumors due to their proximity to optic pathways, the pituitary gland, the hypothalamus, and Willis’ circle [5]. Tumor-induced and treatment-related hypothalamic damage are the major causes of high recurrence and poor prognosis.

Recent studies involving single-nucleus RNA-Seq and spatial transcriptome profiling of ACPs have shown that CAFs are abundant in the ACP microenvironment [25]. Meanwhile, CAFs are found to secrete PROS1 and GAS6, which can activate AXL receptors on the surface of tumor epithelium cells, promoting immune suppression and tumor progression in ACPs [25]. These findings align with our ACP primary cells, which demonstrate CAF features characterized by elevated expression of FSP1, Vimentin and Fibronectin, and were plentiful in the fibrous stroma area. Transcriptomic profiling further revealed significant enrichment of CAF-associated pathways, including extracellular matrix (ECM) organization, collagen fibril assembly and cell-substrate adhesion mechanisms [26,27].

In recent studies, an immortalized ACP cell line with epithelial-like features and the ability to form brain xenografts was established [20]. In the present study, ACP primary cells with CAF features also exhibited tumorigenicity, suggesting a new perspective on the mechanism of ACPs. Tissue and cell heterogeneity were key challenges in ACP research, and the ACP primary cells with CAF features made it possible to further investigate the intercellular interactions within ACPs.

Furthermore, we observed high expression of Vimentin in both 0913 and 0824 ACP primary cells and tissue sections, which is one marker of epithelial-mesenchymal transition (EMT) [28,29]. GSEA revealed significant upregulation of EMT-related pathways in both 0913 and 0824 primary cells compared to normal brain tissue controls. A previous study has demonstrated that EMT occurred in craniopharyngioma, proofed by the expression of Vimentin and E-cadherin in tumor sections, and associated with tumor recurrence [30]. In our previous proteomics study of ACPs, EMT-related pathways and proteins were found to be upregulated in recurrent ACPs compared with primary ACPs [31]. The aforementioned results suggest that EMT could play a crucial role in ACPs’ development and recurrence by promoting tumor proliferation, migration and invasion [32].

ACP is thought to be driven by missense mutations in CTNNB1 [12]. However, our ACP primary cells exhibited only synonymous mutations in CTNNB1. In a previous study, the CTNNB1 missense mutation in exon 3 was only detected in partial ACP epithelial cells [33]. While CTNNB1 mutations are critical for ACP initiation, they are not essential for the proliferation of tumor epithelium. ACP primary cells were transplanted subcutaneously and formed neoplasms in NCG mice. HE and IHC staining revealed pan-CK-positive epithelial cells that were highly similar to the palisade-like epithelial cell layers in ACP tumor tissues. Therefore, our primary cells with CAF features potentially exhibited oncogenicity, and the absence of CTNNB1 missense mutations does not negate their utility as an ACP PDC model.

We hypothesized that the tumor formation of present primary cells was caused by the occurrence of mesenchymal epithelial transition (MET) in mice. When tumor cells undergo EMT, intercellular adhesion is reduced, and migration and invasion are enhanced, which causes tumor cells to escape from the primary lesion. Subsequently, tumor cells’ reversal shows restoration of the epithelial phenotype, and they regain their adhesion ability through MET under the influence of the microenvironment [34,35,36]. However, this hypothesis necessitates further verification through subsequent experimental studies.

In addition to mutations in CTNNB1, other high-frequency mutations, including mutations in UBE4A, ALDH16A1, ACSL3, COL3A1 and SLC12A8, were detected in both ACP primary cells and tissues. These genes are closely related to the occurrence or suppression of tumors. The ubiquitin ligase UBE4A inhibits prostate cancer progression by targeting interleukin-like EMT inducers [37]. ALDH16A1 and COL3A1 play important roles in regulating the immunosuppressive microenvironment, tumor cell proliferation and EMT process in glioma [38,39]. High ACSL3 expression has been detected in a variety of cancers and is correlated with a poor prognosis in patients with these diseases [40], and ACSL3-mediated fatty acid oxidation is required for TGFβ1-induced EMT and metastasis in colorectal carcinoma [41]. SLC12A8 is highly correlated with the oncogenesis and progression of bladder cancer and promotes the expression of EMT protein markers, including β-catenin, vimentin, snail, and slug, through the JAK/STAT pathway [42,43]. Notably, all the high frequencies of mutations identified above were correlated with the EMT process, indicating that EMT plays a crucial role in ACP development and may be a potential therapeutic strategy for ACPs.

In this study, RNA-seq was performed to investigate the gene transcription differences between 0913 (primary) and 0824 (recurrent) primary cells. Notably, the upregulated Hippo, Wnt and Notch signaling pathways and signaling pathways regulating pluripotency of stem cells were all enriched in 0824 versus 0913 primary cells. All of these pathways regulate cell proliferation, apoptosis, stem cell self-renewal and pluripotency [44,45]. Moreover, the Rap1 signaling pathway, which plays a crucial role in tumor cell migration and invasion, was upregulated [46]. The above pathways revealed the increased stemness, proliferative and metastasis ability of primary cells from recurrent tissue. On the other hand, multiple immune-related pathways were downregulated in the 0824 primary cells. Previous studies have shown that CAFs interact with tumor-infiltrating immune cells via the secretion of various cytokines, growth factors, chemokines and exosomes, consequently shaping an immunosuppressive TME that enables cancer cells to evade immune system surveillance [47].

To sum up, our findings suggest that CAF cells in ACPs may contribute to the tumorigenesis through the following mechanisms: (1) ECM remodeling promotes tumor cell adhesion, migration and invasion; (2) EMT enhances tumor cell plasticity and therapy resistance, while CAF-mediated MET in xenografts recapitulates ACPs’ invasive phenotype; and (3) immunosuppressive microenvironment facilitates tumor progression. These mechanisms also suggest that CAF in ACPs may influence prognosis, and our cell models could facilitate drug screening targeting CAF-ECM interactions (e.g., Fibronectin inhibitors) to improve outcomes.

However, this study has several limitations that warrant further investigation. First, while we have demonstrated the tumorigenic potential of ACP primary cells through subcutaneous xenograft models, future studies should establish orthotopic intracranial models to better recapitulate the tumor microenvironment and validate in situ tumorigenicity. Second, the molecular mechanisms underlying ACP tumorigenesis require more comprehensive elucidation. Third, increasing the sample size or using paired samples from the same patient are needed to fully characterize the molecular distinctions between primary and recurrent ACP cells.

## 5. Conclusions

We successfully isolated and characterized ACP primary cells which obtained several CAF features and could be stably passaged for dozens of generations. The establishment of ACP primary cell models provides a valuable platform that will facilitate mechanistic studies and therapeutic discovery in ACP research.

## Figures and Tables

**Table 2:** Genotyping results of the short tandem repeat (STR) loci of the 0913 and 0824 ACP primary cells.

STR Loci	ACP0913	Database: Lu-138 (Matching Rate: 73.33%)	ACP0824	Database: Yub2503c (Matching Rate: 78.57%)
Amelogenin	X	X	X	X
CSF1PO	11, 13	11, 12	12	10, 12
D2S1338	19, 24		16, 19	
D3S1358	16, 17		15, 18	
D5S818	10	10, 12	10, 11	10, 11
D7S820	8, 11	10, 11	11, 12	11
D8S1179	10, 11		11, 12	
D13S317	8, 11	8, 11	8, 11	11, 12
D16S539	10, 11	11	10, 12	10, 12
D18S51	14		13, 14	
D19S433	13, 15.2		13, 15	
D21S11	30, 31		30, 31	
FGA	22, 24		22	
PentaD	11		8, 12	
PentaE	12, 17		5, 16	
TH01	7, 9	7, 9	7, 9	7, 9
TPOX	8, 11	8, 11	9, 11	9, 11
vWA	18, 19	17, 18	14	17
D6S1043	12, 14		19, 21	
D12S391	19, 21		18, 19	
D2S441	11, 12		12, 14	

**Table 3:** (**a**) WES analysis of the gene mutations in the original tumor tissue from patient 0913 and SNPs of derived 0913 primary cells. (**b**) WES analysis of the gene mutations in the original tumor tissue from patient 0824 and SNPs of derived 0824 primary cells.

(**a**)											
**0913 Tumor Tissue**						**0913 Primary Cells**					
**Gene**	**Position**	**ID**	**HGVS_C**	**HGVS_P**	**Effect-Priority**	**Gene**	**Position**	**ID**	**HGVS_C**	**HGVS_P**	**Effect-Impact**
***CTNNB1***	chr3	rs121913413	exon3: c.122C > T exon4: c.101C > T	p.Thr41Ile p.Thr34Ile	Missense-High	***CTNNB1***	/	/	/	/	/
***ACSL3***	chr2	rs145194965	exon5: c.725C > T exon6: c.725C > T exon7: c.725C > T	p.Pro242Leu	Missense-High	***ACSL3***	chr2	rs145194965	c.725C > T c.269C > T	p.Pro242Leu p.Pro90Leu	Missense- Moderate
***ALDH16A1***	chr19	rs759657072	exon13: c.1642C > T exon14: c.1795C > T	p.Leu548Leu p.Leu599Leu	Synonymous- Low	***ALDH16A1***	chr19	rs759657072	c.1642C > T c.1795C > T c.1306C > T	p.Leu548Leu p.Leu599Leu p.Leu436Leu	Synonymous- Low
***COL3A1***	chr2	rs1800255	exon30: c.2092G > A	p.Ala698Thr	Missense-Low	***COL3A1***	chr2	rs1800255	c.2092G > A	p.Ala698Thr	Missense- Moderate
	chr2	rs1516446	exon50: c.4059T > G	p.His1353Gln	Missense-Low		chr2	rs1516446	c.4059T > G c.3150T > G	p.His1353Gln p.His1050Gln	Missense- Moderate
(**b**)											
**0824 Tumor Tissue**						**0824 Primary Cells**					
**Gene**	**Position**	**ID**	**HGVS_C**	**HGVS_P**	**Effect-Priority**	**Gene**	**Position**	**ID**	**HGVS_C**	**HGVS_P**	**Effect-Impact**
***CTNNB1***	chr3	rs121913399	exon3: c.100G > A exon4: c.79G > A	p.Gly34Arg p.Gly27Arg	Missense-High	***CTNNB1***	chr3	rs2293303	c.2340C > T	p.Asp780Asp	Synonymous- Low
	chr3	rs2293303	exon15: c.2340C > T	p.Asp780Asp	Synonymous- Low						
***UBE4A***	chr11	rs782399567	exon16: c.2552A > G exon16: c.2573A > G	p.Asn851Ser p.Asn858Ser	Missense-High	***UBE4A***	chr11	rs782399567	c.2552A > G c.2573A > G c.968A > G	p.Asn851Ser p.Asn858Ser p.Asn323Ser	Missense- Moderate
***ALDH16A1***	chr19	rs766834756	exon1: c.76C > A	p.His26Asn	Missense-High	***ALDH16A1***	chr19	rs766834756	c.76C > A	p.His26Asn	Missense- Moderate
	chr19	rs1320303	exon6: c.679C > G	p.Leu227Val	Missense-Low		chr19	rs1320303	c.679C > G c.190C > G	p.Leu227Val p.Leu64Val	Missense- Moderate
***SLC12A8***	chr3	rs761900953	exon9: c.1294C > T exon10: c.1294C > T	p.His432Tyr p.His432Tyr	Missense-High	***SLC12A8***	chr3	rs2981482	c.1991G > A c.1394G > A	p.Arg664Gln p.Arg465Gln	Missense- Moderate
	chr3	rs2981482	exon13: c.1991G > A exon14: c.1991G > A	p.Arg664Gln	Missense-Low						

## Data Availability

The data supporting the conclusions of this article are available from the corresponding author on reasonable request.

## Supplementary Materials

The following supporting information can be downloaded at: https://www.mdpi.com/article/10.3390/biomedicines13040912/s1, Document S1: WES methods. Refs. [48,49,50,51,52,53,54,55,56,57,58,59,60,61,62,63,64,65,66,67,68,69,70,71,72,73,74,75,76,77,78,79,80] are cited in Supplementary Materials file.

## Abbreviations

The following abbreviations are used in this manuscript:

ACP	Adamantinomatous craniopharyngioma
STR	Short tandem repeat
WES	Whole exome sequencing
CAF	Cancer-associated fibroblast
GTR	Gross total resection
PDC	Patient-derived tumor cell
PDO	Patient-derived tumor cell organ
PDX	Patient-derived tumor xenograft
PBS	Phosphate-buffered saline
TCGA	The cancer genome atlas
GO	Gene ontology
KEGG	Kyoto encyclopedia of genes and genomes
GSEA	Gene set enrichment analysis
DEG	Differential expressed gene
SNP	Single-nucleotide polymorphism
SMG	Significantly mutated gene
ECM	Extracellular matrix
EMT	Epithelial-mesenchymal transition
MET	Mesenchymal-epithelial transition
