# Supplementary material for: Isolation and Characterization of the Adamantinomatous Craniopharyngioma Primary Cells with Cancer-Associated Fibroblast Features
Source: Biomedicines. 2025 Apr 9;13(4):912. doi: 10.3390/biomedicines13040912 (PMC12025058; doi:10.3390/biomedicines13040912)
Supplement: Supplementary file 1 [file biomedicines-13-00912-s001.zip › biomedicines-3535673-supplementary.pdf]

Supplementary Material

Method of WES

1 Experimental Procedure

1.1 Sample Quality Control

Please refer to the QC report for methods of sample quality control.

1.2 Library Preparation

The exome sequences were efficiently enriched from 0.4 μg genomic DNA using Agilent SureSelect Human All Exon V6 (Agilent USA, Catalog #: 5190- 8864)/Agilent SureSelectXT Mouse All Exon library (Agilent, USA, Catalong #: 5190-4643) according to the manufacturer's protocol. First, qualified genomic DNA was randomly fragmented to an average size of 180-280bp by Covaris LE220R-plus (Covaris,USA). Remaining overhangs were converted into blunt ends via exonuclease polymerase activities. Second, DNA fragments were end repaired and phosphorylated, followed by A-tailing and ligation at the 3'ends with paired-end adapters. DNA fragments with ligated adapter molecules on both ends were selectively enriched in a PCR reaction. After PCR reaction, libraries hybridize with liquid phase with biotin labeled probe, then use magnetic beads with streptomycin to capture the exons of genes. Captured libraries were enriched in a PCR reaction to add index tags to prepare for sequencing. Products were purified using AMPure XP system (Beckman Coulter, Beverly, USA), libraries were analyzed for size distribution by Agilent 5400 system (AATI) (Agilent, USA) and quantified by real-time PCR (Life Technologies, USA) (1.5 nM).

The qualified libraries were pooled and sequenced on Illumina platforms with PE150 strategy in Novogene Bioinformatics Technology Co., Ltd (Beijing, China), according to effective library concentration and the data amount required.

2. Bioinformatics Analysis Pipeline

2.1 Raw data

The original fluorescence image files obtained from Illumina platform were transformed to short reads (Raw data) by base calling, and these short reads were recorded in the

FASTQ format, which contains sequence information and corresponding sequencing quality information.

2.2 Data Quality Control

It was the sequence artifacts, including reads containing adapter contamination, low-quality nucleotides and unrecognizable nucleotide (N), that undoubtedly set the barrier for the subsequent reliable bioinformatics analysis. Hence, quality control is an essential step to mitigate these obstacles and could be applied to guarantee meaningful downstream analysis.

The steps of data processing were as follows:

(1) Discardapairofreadsifeitheroneofthemcontainsadapter contamination (>10 nucleotides aligned to the adapter, allowing ≤ 10% mismatches);

(2) Discardapairofreadsifmorethan10%ofbasesareuncertainineitherone of the read;

(3) Discardapairofreadsiftheproportionoflowquality(Phredquality<5) bases is over 50% in either one of the read.

Total reads number, raw data, error rate, and percentage of reads with Q30 (the percent of bases with Phred-scaled quality scores greater than 30) were calculated and summarized. After which, filtered reads were used as clean data for subsequent analysis.

2.3 Sequence Alignment

Clean data were mapped to the reference genome by Burrows Wheeler Aligner (BWA) software (Li et al., 2018) and Samblaster (Faust et al., 2014) to generate a BAM file. Subsequently, Sambamba (Tarasov et al., 2015) was used to sort BAM files and mark duplicate reads according to chromosome position.

2.4 Variant Detection

2.4.1 Germline Mutation Detection

SAMtools (Li et al., 2009) was used to call germline SNP and InDel. The filter parameters of SNP and InDel are shown as follows: QUAL≥20;DP≥4;MQ≥30.

2.4.2 Somatic Mutation Detection (Only for normal-tumor paired samples)

Somatic mutation detection is commonly applied to normal and tumor- paired samples. The somatic SNV was detected by MuTect (Cibulskis et al., 2013), while the somatic InDel was identified by Strelka (Saunders et al., 2012). Control-FREEC (Boeva et al., 2012) was used to detect somatic CNV. The parameter with window = 0 was set as officially recommended in the config file for somatic CNV detection.

2.5 Annotation

ANNOVAR (Wang et al., 2010) was used to perform variant annotation. Annotation contents refer to protein-coding changes, genomic regions affected by the variants, allele frequency, deleterious prediction, etc. The main databases used were as follows:

Genes and regions annotation

RefSeq (O'Leary et al., 2016) and Gencode (Frankish et al., 2021) databases were used to find genomic regions affected by variants and possible changes in the protein. We annotated the features of the genomic regions affected by the variants, such as cytoband, small RNA, conserved mammalian microRNA regulatory target sites, conservative regions of vertebrates, transcription factor binding sites, repeats, etc.

Databases with frequency annotation

The established databases, such as 1000 Genomes (1000 Genomes Project Consortium) (Abecasis et al., 2012), Exome Aggregation Consortium (ExAC) (Kobayashi et al., 2017), Genome Aggregation Database (gnomAD) (Pio et al., 2021) and exome sequencing project (ESP), were all used to find alternative allele frequencies in the populations that were reported. There are a great number of common polymorphism sites in the human population, while many deleterious variants are rare or of low frequency.

Databases and scores with conservative and deleterious annotation

SIFT (Ng et al., 2003), PolyPhen (Adzhubei et al., 2013), MutationAssessor (Reva et al., 2011), LRT (Chun et al., 2009), and CADD (Rentzsch et al., 2019) scores were used to predict the deleterious mutations. GERP++ (Huber et al., 2020) scores were used to evaluate the conservation of mutations. SIFT, Polyphen2, MutationTaster (Steinhaus et al., 2021), LRT, MutationAssessor and FATHMM (Shihab et al., 2013) were all used to predict whether an amino acid substitution affected protein function. SiPhy (Garber et al., 2009), phyloP (Pollard et al., 2010), GERP++ and CADD were all used to predict the conservative level of the site. It should be noted that the conservation scores only consider the conservative level at the current site, but not the one involved in the nucleotide identity. Therefore, synonymous and non-synonymous variants at the same site will have the same scores. These scores are used for finding

functionally important sites, which means that variants that confer increased susceptibility would score well.

Databases with cancer and disease-related annotation

dbSNP (Sherry et al., 2001), COSMIC (Tate et al., 2019), OMIM (Hamosh et al., 2005), GWAS Catalog (Buniello et al., 2019) and HGMD (Stenson et al., 2020) were used to find reported information of the variants.

Databases with functional and pathway annotation.

Gene Ontology (Lee et al., 2004), KEGG (Kanehisa et al., 2000), Reactome (Jassal et al., 2020) and PID (Schaefer et al., 2009) databases were applied to provide functional or pathway annotation.

3 References

Abecasis GR, Auton A, et al. An integrated map of genetic variation from 1,092 human genomes.Nature. 2012;491(7422):56-65. doi:10.1038/nature11632 (1000 Genomes)

Adzhubei I, Jordan DM, Sunyaev SR. Predicting functional effect of human missense mutations using PolyPhen-2.Curr Protoc Hum Genet. 2013;Chapter 7:Unit7.20. doi:10.1002/0471142905.hg0720s76 (PolyPhen)

Boeva V, Popova T, Bleakley K, et al. Control-FREEC: a tool for assessing copy number and allelic content using next-generation sequencing data. Bioinformatics. 2012;28(3):423-425. doi:10.1093/bioinformatics/btr670 (Control-FREEC)

Buniello A, MacArthur JAL, Cerezo M, et al. The NHGRI-EBI GWAS Catalog of published genome-wide association studies, targeted arrays and summary statistics 2019. Nucleic Acids Res. 2019;47(D1):D1005-D1012. doi:10.1093/nar/gky1120 (GWAS Catalog)

Chun S, Fay JC. Identification of deleterious mutations within three human genomes. Genome Res. 2009;19(9):1553-1561. doi:10.1101/gr.092619.109 (LRT)

Cibulskis K, Lawrence MS, Carter SL, et al. Sensitive detection of somatic point mutations in impure and heterogeneous cancer samples.Nat Biotechnol. 2013;31(3):213-219. doi:10.1038/nbt.2514 (MuTect)

Faust GG, Hall IM. SAMBLASTER: fast duplicate marking and structural variant read extraction. Bioinformatics. 2014;30(17):2503-2505. doi:10.1093/bioinformatics/btu314 (Samblaster)

Frankish A, Diekhans M, Jungreis I, et al. GENCODE 2021.Nucleic Acids Res. 2021;49(D1):D916-D923. doi:10.1093/nar/gkaa1087 (GENCODE)

Garber M, Guttman M, Clamp M, Zody MC, Friedman N, Xie X. Identifying novel constrained elements by exploiting biased substitution patterns.Bioinformatics. 2009;25(12):i54-i62. doi:10.1093/bioinformatics/btp190 (SiPhy)

Hamosh A, Scott AF, Amberger JS, Bocchini CA, McKusick VA. Online Mendelian Inheritance in Man (OMIM), a knowledgebase of human genes and genetic disorders. Nucleic Acids Res. 2005;33(Database issue):D514-D517. doi:10.1093/nar/gki033 (OMIM)

Harris MA, Clark J, Ireland A, et al. The Gene Ontology (GO) database and informatics resource.Nucleic Acids Res. 2004;32(Database issue):D258-D261. doi:10.1093/nar/gkh036 (GO)

Huber CD, Kim BY, Lohmueller KE. Population genetic models of GERP scores suggest pervasive turnover of constrained sites across mammalian evolution. PLoS Genet. 2020;16(5):e1008827. Published 2020 May 29. doi:10.1371/journal.pgen.1008827 (GERP)

Jassal B, Matthews L, Viteri G, et al. The reactome pathway knowledgebase. Nucleic Acids Res. 2020;48(D1):D498-D503. doi:10.1093/nar/gkz1031 (Reactome)

Kanehisa M, Goto S. KEGG: kyoto encyclopedia of genes and genomes. Nucleic Acids Res. 2000;28(1):27-30. doi:10.1093/nar/28.1.27 (KEGG PATHWAY)

Kent WJ, Sugnet CW, Furey TS, et al. The human genome browser at UCSC. Genome Res. 2002;12(6):996-1006. doi:10.1101/gr.229102 (UCSC)

Kobayashi Y, Yang S, Nykamp K, Garcia J, Lincoln SE, Topper SE. Pathogenic variant burden in the ExAC database: an empirical approach to evaluating population data for clinical variant interpretation. Genome Med. 2017;9(1):13. Published 2017 Feb 6. doi:10.1186/s13073-017-0403-7 (ExAc)

Li H, Durbin R. Fast and accurate long-read alignment with Burrows-Wheeler transform.Bioinformatics. 2010;26(5):589-595. doi:10.1093/bioinformatics/btp698 (BWA_MEM)

Li H, Handsaker B, Wysoker A, et al. The Sequence Alignment/Map format and SAMtools. Bioinformatics. 2009;25(16):2078-2079. doi:10.1093/bioinformatics/btp352 (SAMtools)

Ng PC, Henikoff S. SIFT: Predicting amino acid changes that affect protein function. Nucleic Acids Res. 2003;31(13):3812-3814. doi:10.1093/nar/gkg509 (SIFT)

O'Leary NA, Wright MW, Brister JR, et al. Reference sequence (RefSeq) database at NCBI: current status, taxonomic expansion, and functional annotation.Nucleic Acids Res. 2016;44(D1):D733-D745. doi:10.1093/nar/gkv1189 (RefSeq)

Pio MG, Siffo S, Scheps KG, et al. Curating the gnomAD database: Report of novel variants in the thyrogobulin gene using in silico bioinformatics algorithms. Mol Cell Endocrinol. 2021;534:111359. doi:10.1016/j.mce.2021.111359 (gnomAD)

Pollard KS, Hubisz MJ, Rosenbloom KR, Siepel A. Detection of nonneutral substitution rates on mammalian phylogenies. Genome Res. 2010;20(1):110-121. doi:10.1101/gr.097857.109 (phyloP)

Rausch T, Zichner T, Schlattl A, Stütz AM, Benes V, Korbel JO. Boeva V, Popova T, Bleakley K, et al. Control-FREEC: a tool for assessing copy number and allelic content using next-generation sequencing data. Bioinformatics. 2012;28(3):423-425. doi:10.1093/bioinformatics/btr670 (Control-FREEC)

Rentzsch P, Witten D, Cooper GM, Shendure J, Kircher M. CADD: predicting the deleteriousness of variants throughout the human genome.Nucleic Acids Res. 2019;47(D1):D886-D894. doi:10.1093/nar/gky1016 (CADD)

Reva B, Antipin Y, Sander C. Predicting the functional impact of protein mutations: application to cancer genomics.Nucleic Acids Res. 2011;39(17):e118. doi:10.1093/nar/gkr407 (MutationAssessor)

Saunders CT, Wong WS, Swamy S, Becq J, Murray LJ, Cheetham RK. Strelka: accurate somatic small-variant calling from sequenced tumor-normal sample pairs. Bioinformatics. 2012;28(14):1811-1817. doi:10.1093/bioinformatics/bts271 (Strelka)

Schaefer CF, Anthony K, Krupa S, et al. PID: the Pathway Interaction Database. Nucleic Acids Res. 2009;37(Database issue):D674-D679. doi:10.1093/nar/gkn653 (PID)

Sherry ST, Ward MH, Kholodov M, et al. dbSNP: the NCBI database of genetic variation. Nucleic Acids Res. 2001;29(1):308-311. doi:10.1093/nar/29.1.308 (dbSNP)

Shihab HA, Gough J, Cooper DN, et al. Predicting the functional, molecular, and phenotypic consequences of amino acid substitutions using hidden Markov models. Hum Mutat. 2013;34(1):57-65. doi:10.1002/humu.22225 (FATHMM)

Steinhaus R, Proft S, Schuelke M, Cooper DN, Schwarz JM, Seelow D. MutationTaster2021. Nucleic Acids Res. 2021;49(W1):W446-W451. doi:10.1093/nar/gkab266 (MutationTaster)

Stenson PD, Mort M, Ball EV, et al. The Human Gene Mutation Database (HGMD®): optimizing its use in a clinical diagnostic or research setting.Hum Genet. 2020;139(10):1197-1207. doi:10.1007/s00439-020-02199-3 (HGMD)

Tarasov A, Vilella AJ, Cuppen E, Nijman IJ, Prins P. Sambamba: fast processing of NGS alignment formats. Bioinformatics. 2015;31(12):2032-2034. doi:10.1093/bioinformatics/btv098 (Sambamba)

Tate JG, Bamford S, Jubb HC, et al. COSMIC: the Catalogue Of Somatic Mutations In Cancer.Nucleic Acids Res. 2019;47(D1):D941-D947. doi:10.1093/nar/gky1015 (COSMIC)

Wang K, Li M, Hakonarson H. ANNOVAR: functional annotation of genetic variants from high-throughput sequencing data.Nucleic Acids Res. 2010;38(16):e164. doi:10.1093/nar/gkq603 (ANNOVAR)
